# Assessment of Symptoms and Risk Factors as a Screening Tool of Bacterial Vaginosis Among Reproductive Age-Group Females in West Bengal, India

**DOI:** 10.7759/cureus.46310

**Published:** 2023-10-01

**Authors:** Anzum Nuzhad, Bappaditya Ghosh, Subhas Chandra Jana

**Affiliations:** 1 Microbiology, Raiganj University, Raiganj, IND; 2 Zoology, North Bengal University, Darjeeling, IND

**Keywords:** valvovaginal candidiasis, screening tool, syndromic diagnosis, risk factors for bv, bacterial vaginosis (bv)

## Abstract

Background

Bacterial vaginosis (BV) is a ubiquitous vaginal discomfort and has overlapping symptoms with other reproductive tract infections. The World Health Organization suggested a symptomatic approach for diagnosing BV with insufficient laboratory setup. However, due to symptom overlap, BV is often misled and ends up with injudicious drug application.

Objective

The study aims to identify the most relevant symptoms and behavioral risk factors associated with BV in tertiary healthcare settings in West Bengal, India. It also seeks to develop a scoring system based on clinical symptoms to screen for BV, especially when laboratory facilities are limited.

Methodology

The study is a retrospective case-control study involving 95 women of reproductive age. It employs both univariate and multivariate binary logistic regression models to identify risk factors and symptoms associated with BV. The study also compares these clinical symptoms with laboratory tests (Amsel’s test) and attempts to create a scoring system for BV diagnosis.

Key Findings

Good menstrual hygiene and condom use were identified as key behavioral practices reducing the risk of BV. Four clinical symptoms, like malodor (*P *= 0.007), lower abdominal pain (*P *= 0.015), abnormal vaginal discharge (*P *= 0.071), and painful intercourse (*P *= 0.08), were identified as notable predictors. Based on these four symptoms, the scoring system showed a sensitivity of 88.2%, a specificity of 67.25%, and an overall accuracy of 74.7%. An additional diagnosis of vulvovaginal candidiasis (VVC) revealed that the odds of malodor (*P*= 0.006) and burning sensation (*P*= 0.011) increased significantly during co-infection.

## Introduction

Bacterial vaginosis (BV) is one of the most common reproductive tract infections in India. It is the prevalent cause of abnormal vaginal discharge among reproductive age-group females [1]. BV is a well-known vaginal dysbiosis with the replacement of beneficial lactic acid bacteria, e.g., lactobacilli with anaerobic bacteria (e.g., *Gardnerella vaginalis*, *Atopobium vaginae*, and *Mycoplasma hominis*) [2]. Generally, it does not show any sign of inflammation but can potentially cause adverse pregnancy and peri-natal outcomes. BV can increase the risk of post-abortion pelvic inflammatory disease, post-hysterectomy vaginal cuff infection, chorioamnionitis, pre-term labor, and mid-trimester miscarriages [3]. With dysbiosis and a broken vaginal first line of defense, BV increases the risk of the acquisition of various opportunistic pathogens, including the human papillomavirus (HPV) [4].

Amsel’s test is good for diagnosing symptomatic BV, where BV can be detected based on three out of four clinical criteria. Among the four criteria for Amsel’s test, amine test and microscopic observation required a good bedside setting and an apparent sterile environment [5]. The World Health Organization (WHO) recommended a symptomatic approach for the centers where advanced facilities are not available [6]. Only a few tertiary healthcare settings in India have advanced types of BV testing setups. The absence of the necessary facilities and the overwhelming patient load prevented out-patient departments from managing enough time for testing. They mostly rely on the symptomatic approach and patients’ clinical history to diagnose. However, those symptoms are often shared with reproductive tract infections other than BV [7]. With similar symptoms, vulvovaginal candidiasis (VVC) and BV frequently co-occur [8]. Therefore, it’s crucial to comprehend the primary signs and how frequently they occur during mixed infection with VVC. Even though there are several reports of BV from India [9-12], only a small number of them contain in-depth statistical studies. In particular, neither West Bengal nor the rest of northeastern India have any.

In the above scenario, re-evaluation of symptoms and assessment of the most statistically relevant and significant symptoms for BV are required for maximum diagnostic success in such rural and semi-urban areas of India. The study statistically analyzed the most relevant symptoms for BV along with associated behavioral risk factors in tertiary healthcare settings in the urban area of West Bengal. It also tried to comment on the co-occurrence of BV with VVC with a special emphasis on symptom overlap. Finally, we aimed to make a BV screening scoring tool incorporating significant symptoms to identify BV-positive cases.

## Materials and methods

Study design

This research was a retrospective case-control study among reproductive age-group females. Ethical approval was obtained from the Institutional Ethics Committee of Raiganj Government Medical College & Hospital, Raiganj, India (RGMC&H) (Proposal No. RGMCH/IEC/2020/02, No. IEC.11/2020 dated 10/10/2020). The research was conducted from April to December 2021 at the Department of Microbiology, Raiganj University, with the co-operation of RGMC&H. The case group included the patients who were diagnosed with BV positive, and the control group was the patients who were diagnosed with negative for BV.

Patient and public involvement statement

The patients who were visiting the outpatient department (OPD) of gynecology and obstetrics, RGMC&H, under the reproductive age-group (18-45 years), had been enrolled in the study. A written consent form was signed by each participant. Each enrolled respondent had the right to withdraw at any point in the study if they thought to leave. The researchers ensured the proper confidentiality of the participant’s information. The participants were then interviewed by the research team on a previously standardized information sheet (Appendix Table 5) to obtain socio-demographic, menstrual hygienic behavior, gravida status, case history, and present symptoms data of the participants. The number of patients involved in the study was directly proportional to the result and its effects. During their OPD visit, patients’ involvement was limited to an interview and a single vaginal swab sample.

Selection of subjects

This study limited the patient number by selecting the following exclusion and inclusion criteria for participation.

Inclusion Criteria

The inclusion criteria include reproductive age-group (18-45 years) women (both pregnant and non-pregnant).

Exclusion Criteria

The exclusion criteria include women below 18 years and menopausal females, women who have received antibiotics within the past two weeks, women having sexual intercourse within the past two days or taking spermicides, menstruating females, women who have serious gynecology and obstetrics history, and immunocompromised, AIDS, and COVID-19 patients.

Collection of samples

Following the Helsinki Declaration on research bioethics, only the willing patients participated in this program by providing their written consent [13]. All of the participants were first clinically investigated by the attending gynecologist, and their findings were recorded. The vaginal swab was collected, and the vaginal pH was recorded by the attending gynecologist with the help of the nursing staff. The samples were sent to the microbiology laboratory within two hours in the labeled tubes using normal saline as a medium.

Laboratory testing

All the vaginal samples were tested blindly in the microbiology laboratory. The pH was tested by using pH paper following reference colors for pH detection. The presence of clue cells, yeast buds, and any motile protozoan findings was recorded in wet-mount cytology under a 1000× light microscope and followed by gram staining (Appendix Figures 2, 3). One drop of 10% KOH solution was added to one drop of vaginal aspirate, and the release of any fishy odor due to amine was recorded. BV diagnosis was made by following four parameters, i.e., vaginal pH > 4.5, thin homogenous abnormal vaginal discharge, “fishy odor” after applying 10% KOH solution (whiff test), and presence of >20% clue cells on wet mount. The sample was declared positive when three or all four criteria were matched [5]. Samples were considered as VVC positive when yeast-bud or pseudo-hyphae were present during the microscopic study [14].

Statistical study

Initially, the data were imported to Microsoft Excel in coded form; then, for statistical analysis, the data were transferred to IBM SPSS Statistics, version 26.0 (IBM Corp., Armonk, NY). Descriptive statistics and logistical regressions were used to identify the risk factors for BV. The variables were tested in the univariate binary logistic regression model, and the determinants with significant P-value (P ≤ 0.25) [15], including confounders and the case-reported suspected risk factors, were further analyzed by multivariate analysis with BV as the dependent variable. Confounders were explored by comparing the difference between the adjusted odd ratio (AOR) in multivariate analyses and the crude odd ratio (COR) in univariate analyses with a 95% confidence interval (CI) along their respective Crude/adjusted P-values to present the association. A scoring system to predict the BV was implied based on the predictors found significant in the multivariate binary logistic regression model using the backward conditional method considering the stepwise removal probability of 0.15. The receiver’s operating characteristic (ROC) curve analysis was performed based on the scoring system, and the sensitivity, specificity, and overall accuracy were also estimated. The association of symptoms with BV during co-infection with VVC was tested using individual symptoms as a dependent variable and BV and VVC as a categorical variable.

## Results

The present study tried to point out possible risk factors for BV with clinical symptoms. In all, 95 patients participated, including 34 (35.4%) in the case group and 61 in the control group. Table 1 represents the demographic characteristics of patients. No significant association was found in reproductive age, economic status, educational status, irregular menstruation, pregnancy status, occupation, religion, community, gravida status, child death, and previous case history status with BV.

**Table 1:** Demographic characteristics and daily practices of the participants (N = 95) in relation to BV status ^*^P-value ≤ 0.05 APL, above poverty line; BPL, below poverty line; Gen, general; OBC, other backward caste; SC, scheduled castes; ST, scheduled tribes; BV, bacterial vaginosis; COR, crude odd ratio; AOR, adjusted odd ratio

Characteristics (N)	BV = yes	BV = no	COR	AOR	Coefficient (B)
	N (row%)	N (row%)	95% CI	95% CI	
			Crude P-value	Adjusted P-value	
Occupation					
House wife (89)	30 (33.7)	59 (66.3)	1	NA	NA
Worker (6)	4 (66.7)	2 (33.3)	3.933		
			2.085-12.754		
			0.126		
Socioeconomic status					
APL (48)	15 (31.3)	33 (68.8)	1	NA	NA
BPL (47)	19 (40.4)	28 (59.6)	1.493		
			0.642-3.471		
			0.352		
Religion					
Hindu (63)	21 (33.3)	42 (66.7)	0.731	NA	NA
			0.304-1.759		
			0.484		
Muslim (32)	13 (40.6)	19 (59.4)	1		
Community					
Gen (22)	9 (40.9)	13 (59.1)	3.231	NA	NA
			0.128-3.321		
			0.128		
OBC (34)	13 (38.2)	21 (61.8)	2.889		
			0.694-12.023		
			0.145		
SC (22)	9 (40.9)	13 (59.1)	3.231		
			0.714-14.611		
			0.128		
ST (17)	3 (17.6)	14 (82.4)	1		
Reproductive age					
Early = >18 to <35 years (74)	27 (36.5)	47 (63.5)	1	NA	NA
Late = >35 to ≤45 years (21)	7 (33.3)	14 (66.7)	0.87		
			0.313-2.422		
			0.79		
Pregnancy status					
Non-pregnant (51)	21 (41.2)	30 (58.8)	1.669	NA	NA
			0.710-3.924		
			0.24		
Pregnant (44)	13 (29.5)	31 (70.5)	1		
Gestational status					
1st trimester (5)	00 (00)	5 (100)	NA	NA	NA
2nd trimester (18)	4 (22.2)	14 (77.8)			
3rd trimester (21)	9 (42.9)	12 (57.1)			
Child death					
Yes (11)	4 (36.4)	7 (63.6)	1.029	NA	NA
			0.278-3.924		
			0.966		
No (84)	30 (35.7)	54 (64.3)	1		
Gravida					
None (40)	12 (30.0)	28 (70)	1	NA	NA
Uni (33)	11 (33.3)	22 (66.7)	0.122		
			0.146-1.256		
			0.122		
Multiple (22)	11 (50)	11 (50)	0.5		
			0.166-1.510		
			0.219		
Educational status					
Educated (75)	26 (34.7)	49 (65.3)	0.796	NA	NA
			0.289-2.192		
			0.659		
Non-educated (20)	8 (40.0)	12 (60.0)	1		
Menstrual hygiene					
Good (37)	9 (24.3)	28 (75.7)	0.121	0.106	-2.24
			0.026-0.554	0.021-0.531	
			0.007*	0.006*	
Moderate (47)	17 (36.2)	30 (63.8)	0.213	0.151	-1.888
			0.050-0.910	0.032-0.720	
			0.037*	0.018*	
Poor (11)	8 (72.7)	3 (27.3)	1	1	0
Contraceptives					
Condom (12)	1 (8.3)	11 (91.7)	0.165	0.169	-1.775
			0.020-1.364	0.018-1.561	
			0.094	0.117	
Oral pills (22)	11 (50.0)	11 (50.0)	1.81	2.219	0.797
			0.672-4.876	0.777-6.334	
			0.241	0.136	
IUD (2)	1 (50.0)	1 (50.0)	1.81	2.571	0.944
			0.108-30.436	0.149-44.419	
			0.68	0.516	
None (59)	21 (35.6)	38 (64.4)	1	1	0
Vaginal washing					
Commercial product (7)	1 (14.3)	6 (85.7)	0.343	NA	NA
			0.038-3.081		
			0.34		
Soap (36)	16 (44.4)	20 (55.6)	1.647		
			0.686-3.956		
			0.264		
Water (52)	17 (32.7)	35 (67.3)	1		

The results of the univariate analyses revealed a significant connection between maintaining menstrual hygiene and BV (P < 0.05). Maintaining good menstrual hygiene reduces the risk (B = −2.240, P = 0.006), followed by the moderate level (B = −1.888, P = 0.018), both having COR and AOR as <1 for BV at P < 0.05. Although having both the AOR and COR as <1 for using condoms as a contraceptive indicates a lower risk of BV (COR = 0.165 at P = 0.094; AOR = 0.169 and B = −1.775 at P = 0.117) and taking oral pills increases the risk (coefficient = 0.797, P = 0.136) for BV compared to those who don’t use any contraceptives, the P-values were found insignificant. The univariate analysis showed that using soap for vaginal washing increases the risk of BV with odd ratio (OR) = 1.647 and 95% CI = 0.686-3.081 if we consider the P-value = 0.264 (Table 1).

Based on the literature review, vaginal itching, malodor, painful intercourse, burning sensation, lower abdominal pain, and abnormal vaginal discharge were selected as clinical symptoms for vaginitis and further evaluated for findings of laboratory-confirmed BV-positive cases. Individually, all six parameters have the following sensitivity, specificity, positive predictive value, and negative predictive values, which are well represented in Table 2.

**Table 2:** Sensitivity, specificity, PPV, and NPV of six selected parameters PPV, positive predictive value; NPV, negative predictive value

Sl. No	Parameter	Sensitivity	PPV	Specificity	NPV
1	Burning sensation	55.9%	65.5%	83.6%	77.3%
2	Vaginal itching	64.7%	57.9%	73.8%	78.9%
3	Malodor	44.1%	78.9%	93.4%	75.0%
4	Painful intercourse	55.9%	65.5%	83.6%	77.3%
5	Lower abdominal pain	88.2%	55.6%	60.7%	90.2%
6	Abnormal vaginal discharge	97.1%	49.3%	44.3%	96.4%

Abnormal vaginal discharge is the most sensitive symptom (97.1% sensitivity) with the highest negative predictive value (96.4%) among the six symptoms. Meanwhile, malodor has the highest specificity of 93.4% compared to the other five parameters. The univariate analysis showed that all the six above-mentioned symptoms were significantly associated with BV (P < 0.05) with a higher OR for symptomatic patients (Table 3).

**Table 3:** Univariate and multivariate model using different symptoms as predictors for BV positive and risk score to predict BV ^*^P value ≤ 0.05 ^†^Malodor, lower abdominal pain, abnormal vaginal discharge, and painful intercourse were found as significant predictors for BV using the multivariate analysis applying the backward conditioning method and used in the scoring system to predict BV BV, bacterial vaginosis; COR, crude odd ratio; AOR, adjusted odd ratio

Predictors (N)	Category	BV = yes	BV = no	COR	AOR	Coefficient (B)	Score
		N (row%)	N (row%)	95% CI	95% CI		
				Crude P-value	Adjusted P-value		
Vaginal itching	Yes (36)	22 (57.9)	16 (42.1)	5.156	1.957	NA	NA
				2.085-12.754	0.580-6.600		
				0.000*	0.279		
	No (57)	12 (21.1)	45 (78.9)	1	1	0	0
Malodor†	Yes (19)	15 (78.9)	04 (21.1)	11.25	9.073	2.004	2
				3.325-38.069	1.833-44.897		
				0.000*	0.007*		
	No (76)	19 (25.0)	57 (75.0)	1	1	0	0
Painful intercourse†	Yes (29)	19 (65.5)	10 (45.5)	6.46	3.472	0.886	1
				2.478-16.838	0.849-14.200		
				0.000*	0.083		
	No (66)	15 (22.7)	51 (77.3)	1	1	0	0
Burning sensation	Yes (29)	19 (65.5)	10 (34.5)	6.46	0.515	NA	NA
				2.478-16.838	0.106-2.502		
				0.000*	0.411		
	No (66)	15 (22.7)	51 (77.3)	1	1	0	0
Lower abdominal pain†	Yes (54)	30 (55.6)	24 (44.4)	11.562	5.809	1.726	2
				3.614-36.990	1.401-24.084		
				0.000*	0.015*		
	No (41)	04 (9.8)	37 (90.2)	1	1	0	0
Abnormal vaginal discharge†	Yes (67)	33 (49.3)	34 (50.7)	26.206	7.791	2.194	2
				3.365-204.093	0.837-72.539		
				0.002*	0.071		
	No (28)	01 (3.6)	27 (96.4)	1	1	0	0

However, the multivariate binary logistic regression revealed that malodor and lower abdominal pain were found to be significantly related to BV (P < 0.05). In contrast, painful intercourse and abnormal vaginal discharge also showed an association at P < 0.1 (Table 3). Vaginal itching and burning sensation were not found to be associated with BV (P > 0.25). Sensing malodor indicated the highest odds (AOR = 9.073, P = 0.007) for BV (Table 3) and was also found to have the highest value of specificity (Table 3).

**Figure 1:**
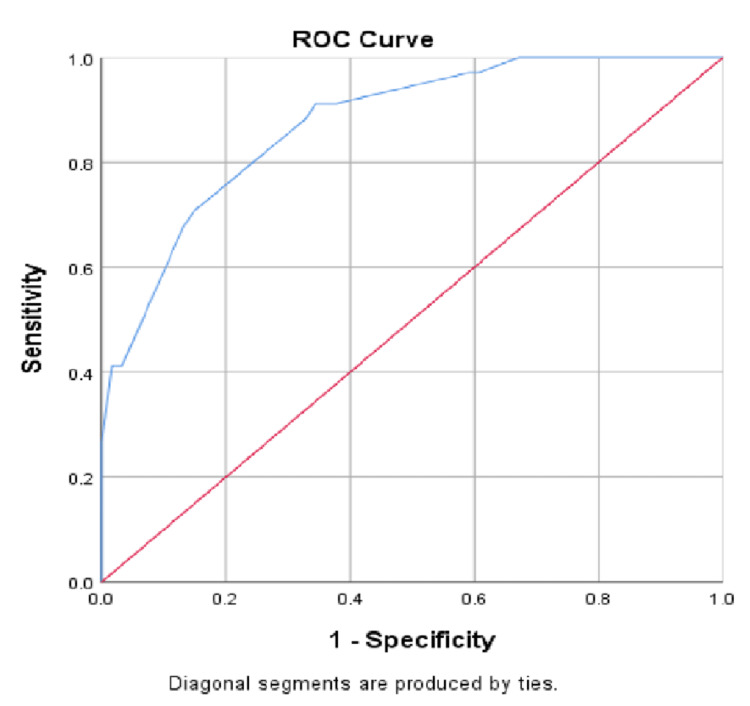
ROC curve to predict BV based on the scores of malodor, lower abdominal pain, abnormal vaginal discharge, and painful intercourse The AUC was 0.874 (standard error = 0.036, 95% CI = 0.804-0.944); a cut-off value of 3 was determined, reflecting a sensitivity of 88.2% and a specificity of 67.2%, with an overall accuracy of 74.7% ROC, receiver’s operating characteristic; BV, bacterial vaginosis; AUC, area under the curve

The abnormal vaginal discharge, with an AOR of 7.791 (P = 0.071), was the most sensitive indication. Moreover, using the multivariate analysis by applying the backward conditioning method, malodor, lower abdominal pain, abnormal vaginal discharge, and painful intercourse were selected as significant predictors for BV and used to implement a scoring system to predict BV (Table 3). The ROC curve analysis revealed that this four-item scoring system set a cut-off score of ≥3 with a specificity of 67.25, a sensitivity of 88.2%, and an overall prediction of 74.7%. The area under the ROC curve was 0.874 (Figure 1).

Malodor, abnormal vaginal discharge, and lower abdominal pain were scored as 2, and painful intercourse was scored as 1 (Table 3). Figure 1 shows the ROC curve to predict BV based on the scores of malodor, lower abdominal pain, abnormal vaginal discharge, and painful intercourse. The AUC was 0.874 (standard error = 0.036, 95% CI = 0.804-0.944). A cut-off value of 3 was determined, reflecting a sensitivity of 88.2% and a specificity of 67.2%, with an overall accuracy of 74.7%.

The study also found that malodor and burning sensation increased with OR = 10.354-14.500 (P = 0.005 and 0.006) and OR = 3.429-8.000 (P = 0.038 and 0.011), respectively, in co-occurrence with yeast infection (VVC). Likewise, lower abdominal pain is more pronounced in BV than in VVC (17.417, 95% CI = 3.454-87.825, P = 0.001) (Table 4).

**Table 4:** Binary logistic regression model showing the relationship between co-infection of BV with VVC and related symptoms (using individual symptoms as a dependent variable and BV and VVC as categorical variables) VVC, vulvovaginal candidiasis; BV, bacterial vaginosis; COR, crude odd ratio

Symptoms	No BV/no yeast	Only yeast	Only BV	BV+ yeast
	COR	COR	COR	COR
		95% CI	95% CI	95% CI
		Crude P-value	Crude P-value	Crude P-value
Vaginal itching	1	0.154	1.636	12.462
		0.038-0.618	0.559-4.790	1.401-110.867
		0.008	0.369	0.024
Malodor	1	1.036	10.357	14.500
		0.136-7.867	1.996-53.754	2.180-96.430
		0.973	0.005	0.006
Painful intercourse	1	0.641	5.833	4.167
		0.161-2.546	1.748-19.468	0.905-19.177
		0.527	0.004	0.067
Burning sensation	1	0.381	3.429	8.000
		0.88-1.640	1.073-10.953	1.626-39.354
		0.195	0.038	0.011
Lower abdominal pain	1	1.056	17.417	6.333
		0.378-2.949	3.454-87.825	1.146-35.008
		0.918	0.001	0.34
Abnormal vaginal discharge	1	0.364	10.954	3 x 10^9^
		0.128-1.034	1.290-93.001	0.000-#
		0.058	0.028	0.364

## Discussion

The present study is going to be the first report from the eastern part of India, particularly in West Bengal, representing lower genital complaints as predictors to screen for BV. The research tried to include pregnant and non-pregnant women in a randomized manner by setting eligibility criteria. This enabled us to concentrate on the general positivity of BV. Malodor, lower abdominal pain, abnormal vaginal discharge, and painful intercourse were the most common symptoms of BV in reproductive age group females. Likewise, menstrual hygiene was found to be the most important healthy practice to keep the vagina free from infections.

Socio-demographic factors like age, gravida status, and education status (lower than high school) also play a significant role in BV. Primary education level and below are the significant predictors of BV. Importantly, pregnant women or women who have multiple gravida status are more susceptible to BV [16]. In this research, we tried to investigate these parameters. The number of patients involved in this kind of study is directly proportional to the result and its effects. However, our research did not yield such relevant results, perhaps due to the limited sample size and diversity.

Menstrual hygiene management is likely to be related to socio-economic status, education, availability of personal space, and recourses provided to the person. The usage of disposable pads over reusable pads is reported to reduce genital infections [17]. Our findings suggested that the odds for BV for women with good and moderate menstrual hygiene are 90% and 85% lower, respectively, than those with poor menstrual hygiene. Intra-vaginal cleaning with soapy water has been linked to BV and the disruption of healthy vaginal flora [18]. Comparing our findings to those of water users, we discovered that using soap to clean the vagina may put women at risk for BV.

BV is not classified as a sexually transmitted disease [19], but it was reported that having protective intercourse can reduce the risk of BV. Condom users were more likely to test negative for BV [1], which is similar to our findings. Our research did not find a negative correlation between BV and oral contraceptives. Although the use of hormonal contraceptives might reduce the acquisition of BV, oral contraceptives increase the chance of an imbalanced vaginal microbiome for BV and VVC [20].

According to the literature review, a symptomatic approach is considered the most realistic and cost-effective method to manage lower genital tract complaints [7,21,22]. Generally, medical practitioners diagnose BV based on symptomatic outcomes, so six self-reported symptoms were used to develop a screening tool for the assessment of BV. This research also points to behavioral daily life practices as a significant factor for acquiring BV. In addition to self-reported symptoms, behavioral daily life practices also help practitioners predict BV. Therefore, behavioral daily life practices were not used for generating a scoring system in this study. As expected, lower genital tract complaints with selected six classic BV symptoms play an excellent role in predicting BV. However, four symptoms (i.e., malodor, lower abdominal pain, abnormal vaginal discharge, and painful intercourse) were selected for scoring due to considerable significant values.

The sensitivity, specificity, and overall accuracy of this study were 88.2%, 67.25%, and 74.7%, respectively. In a similar study, Pastore et al. built ROC with six predictors (i.e., vaginal pH, condom use during pregnancy, antenatal BV, absence of sperm on smear, no history of sexually transmitted diseases, and black race) with both sensitivity and specificity being 77% in their model [23]. However, the present research excludes daily life practices from the predictor; rather, it considers them as one of the risk factors for BV. Our research shows similarities with Nelson et al. [24]. Both of the research use malodor as a predictor. However, Nelson et al. emphasized a maximum specificity of 90.5% [24], whereas our study emphasized maximum sensitivity with justified specificity.

Malodor or “fishy smell” is the primary sign of having BV. This is because the responsible bacteria produce amines such as trimethylamine, cadaverine, and putrescine, which are responsible for the malodor [24]. Malodor has the highest specificity of 93%, just like Gutman et al. [25].

Burning sensation and vaginal itching are mainly associated with an inflammatory state, and as BV is mainly non-inflammatory [19], these symptoms failed to show a significant result to be considered in a scoring system. However, during the co-infection of BV with VVC, the likelihood ratios increased significantly. Klebanoff et al. [26] conducted a symptom-based study on VVC with BV patients who reported 85% vaginal itching in mixed infection cases and 88% with VVC alone. It supported our research findings that having BV alone does not show significant vaginal itching; rather, it increases during co-infection with VVC. Salinas et al. [27] and Das et al. [28] also commented on behavioral risk factors associated with BV and VVC. Salinas et al. addressed contraceptive practices that were remarkably associated with vaginal microbiome structure related to dysbiosis [27]. Das et al. stated that bad sexual practices and poor menstrual hygiene management were associated with lower genital tract infections like BV and VVC [28].

Limitations and future research

Despite having significant results for statistical interpretation, the study should be conducted on a larger population in the future for better and more fruitful analysis. All the predictors (except abnormal vaginal discharge and malodor) were self-reported by the patients, so any miscommunication or shyness about discussing the symptoms might exclude information regarding their current status. The purpose of the research is to build an effective screening tool. It does not claim to confirm the result, so we recommended a final laboratory test for confirmation. This study does not consider the impact of other sexually transmitted infections (STIs) or other potential confounders, and it is retrospective in nature, which limits the ability to establish causality.

## Conclusions

This study statistically evaluates multiple clinical symptoms and behavioral practices that will help screen BV better, where healthcare facilities are poor. Condom usage and proper menstrual hygiene were crucial behavioral practices that lowered the occurrence of BV. It became apparent that four clinical signs, malodor, lower abdominal pain, abnormal vaginal discharge, and painful intercourse, significantly predicted BV. Based on these four symptoms, the scoring system had an overall accuracy of 74.7%, a specificity of 67.25%, and a sensitivity of 88.2%. The study findings revealed that malodor and burning sensations increased significantly during co-infection with VVC. The outcomes could assist with BV screening in primary healthcare settings and alert patients to any unusual symptoms that call for gynecological evaluation. With good menstrual hygiene and safe sex (using a condom as a contraceptive), one could save themselves from BV.

## Figures and Tables

**Figure 2:**
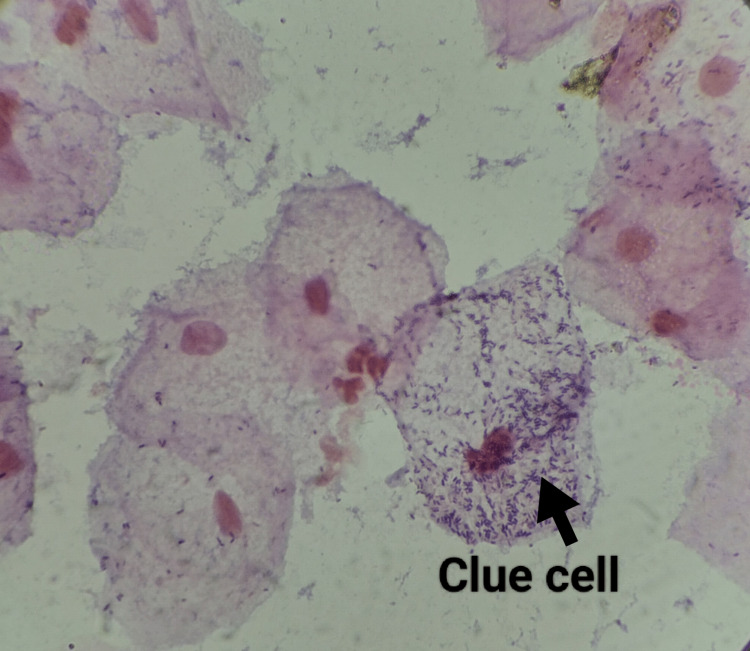
Gram staining images for detection of BV under a light microscope (1000×)

**Figure 3:**
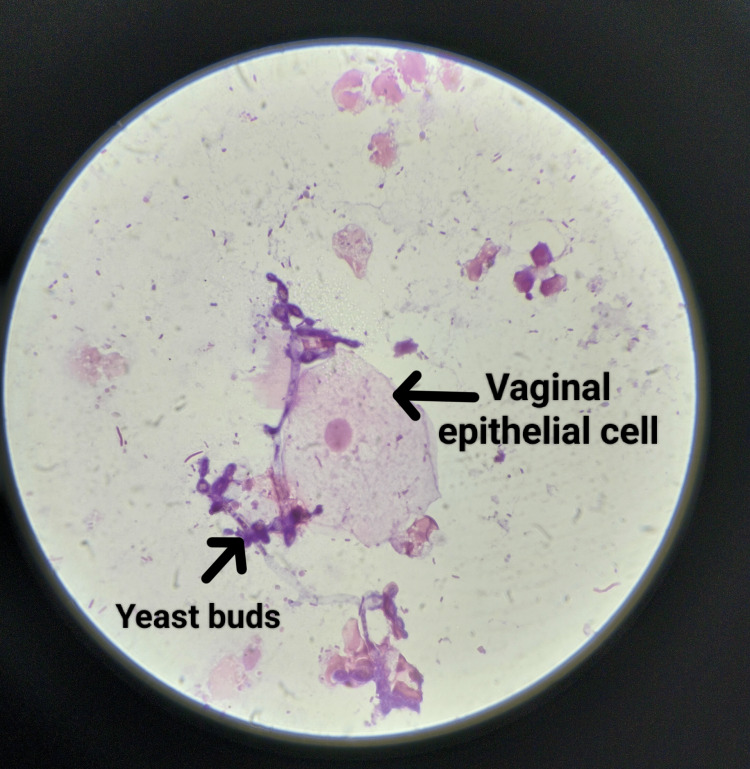
Gram staining images for yeast under a light microscope (1000×)

## Appendices

Supplementary Materials 

**Table 5:** Patient's information sheet

Parameters	Options																															
Age group (in years)	18-23	24-29			30-35					36-40								41-46													47 and above	
Pregnancy status	Pregnant									Non-pregnant																						
Gestation period in months (if applicable)	4	5			6					7								8					9								10	
No. of delivery	Not applicable	1			2					3								4					5								Above	
Previous obstetric history	Normal delivery	Cesarean delivery						Premature delivery						Spontaneous abortion								Still birth 1. Intra-uterine death 2. Death during/after delivery										
No. of child death												Cause of death:																				
Case of infertility	Yes											No																				
Breast feeding	Yes											No																				
Clinical symptoms (if any)	Whitish/yellowish discharge						Itching										Fishy smell										Painful intercourse					
Case of recurrent infection within one year	Yes																No															
Socio economic status	BPL											APL																				
Occupation	Employed/self employed				House wife											Student												Others				
Exercise	Daily				Weekly/monthly														Never													
Diet habit	Pure vegetarian									Non-vegetarian													Fastfoods									
Addictions	Drinking Alcohol Coffee Tea			Smoking						Paan										Tobacco										Others(Drugs)		
Taking of health supplements (please mention brand name if applicable)	Non-steroidal			Steroidal						Probiotics										Ayurveda										Homeopathy		
Awareness about personal hygiene and menarche	Yes (please inform the source)											No																				
Personal hygiene	Poor						Moderate					Good													Excellent							
Access of water for ablution and bathing	In your own house														Outside home																	
Types of absorbents	Disposable														Re-usable																	
No. of changes/day	3 times					Twice															Once											
Place where absorbent is change	Outside					At private room															At household toilet											
Frequency of washing during menstruation	Twice					Once a day															Only 1^st^ day											
Way of washing yourself during menstruation	Soap and water												Water only													Other commercial product						
Menstrual duration	1-4 days				5-9 days										10 or above														Irregular			
Educational qualification	Uneducated		Below class 8						10^th^ class			12^th^ class										Graduate							Post-graduate			
Marital status	Unmarried				Married							Widow												Divorced								
No. of sexual partners	1									2												Multiple										
Use of contraceptives	Pills/tablets				Condom																	Others										
Religion	Hindu				Muslim							Christian												Others								
Previous case history (if applicable)	UTI				PID							PCOD												RTI								
Family history (if applicable)	Breast cancer/cyst				Cervical cancer							Uterine cancer/cyst												Still birth								Ovarian cancer
Resistance to infection	Frequently cough and cold										Normally resistant to infection																					
